# IQ-TREE 3: phylogenomic inference software using complex evolutionary models

**DOI:** 10.1093/molbev/msag117

**Published:** 2026-05-05

**Authors:** Thomas K F Wong, Nhan Ly-Trong, Huaiyan Ren, Piyumal Demotte, Hector Baños, Andrew J Roger, Edward Susko, Chris Bielow, Nicola De Maio, Nick Goldman, Matthew W Hahn, Mario dos Reis, Le Sy Vinh, Gavin Huttley, Robert Lanfear, Bui Quang Minh

**Affiliations:** School of Computing, College of Systems and Society, Australian National University, Canberra, ACT, Australia; Research School of Biology, College of Science and Medicine, Australian National University, Canberra, ACT, Australia; School of Computing, College of Systems and Society, Australian National University, Canberra, ACT, Australia; School of Computing, College of Systems and Society, Australian National University, Canberra, ACT, Australia; School of Computing, College of Systems and Society, Australian National University, Canberra, ACT, Australia; Department of Mathematics, California State University San Bernardino, San Bernardino, CA, USA; Department of Biochemistry and Molecular Biology, Faculty of Medicine, Dalhousie University, Halifax, NS, Canada; Department of Biochemistry and Molecular Biology, Faculty of Medicine, Dalhousie University, Halifax, NS, Canada; Department of Mathematics and Statistics, Faculty of Science, Dalhousie University, Halifax, NS, Canada; Bioinformatics Solution Center, Freie Universität Berlin, Berlin 14195, Germany; European Molecular Biology Laboratory, European Bioinformatics Institute (EMBL-EBI), Hinxton, Cambridgeshire, UK; European Molecular Biology Laboratory, European Bioinformatics Institute (EMBL-EBI), Hinxton, Cambridgeshire, UK; Department of Biology and Department of Computer Science, Indiana University, Bloomington, IN 47405, USA; School of Biological and Behavioural Sciences, Queen Mary University of London, London E1 4NS, UK; University of Engineering and Technology, Vietnam National University, Hanoi, Vietnam; Research School of Biology, College of Science and Medicine, Australian National University, Canberra, ACT, Australia; Research School of Biology, College of Science and Medicine, Australian National University, Canberra, ACT, Australia; School of Computing, College of Systems and Society, Australian National University, Canberra, ACT, Australia

**Keywords:** phylogenetic software, phylogenomic software, maximum likelihood, mixture model, concordance factor, sequence simulator

## Abstract

IQ-TREE (https://iqtree.github.io/) is a widely used open-source software tool for efficiently inferring phylogenetic trees under maximum likelihood. Here, we present IQ-TREE version 3, the third major release of the software. IQ-TREE 3 significantly extends version 2 with new features, including mixture models as an alternative to partitioned models, gene and site concordance factors to quantify discordance between genomic regions, integration with phylogenomic divergence time estimation, and a fully featured sequence simulator. The IQ-TREE 3 source code is available at https://github.com/iqtree/iqtree3.

## Introduction

IQ-TREE (Nguyen et al. 2015; Minh et al. 2020b) is a popular open-source software package for phylogenetic and phylogenomic analysis using a likelihood framework. IQ-TREE has been used by thousands of researchers and practitioners to understand evolution and biodiversity across many species and time scales. These studies range from inferring ancient relationships within Bacteria (Parks et al. 2018; Wehbi et al. 2024), Archaea (Alves et al. 2018; Dombrowski et al. 2020), and eukaryotes (Burki et al. 2016; Williamson et al. 2025); to more recent divergences among plants (Puttick et al. 2018), animals (Redmond and McLysaght 2021), birds (Stiller et al. 2024), gum trees (Crisp et al. 2024), and primates (Vanderpool et al. 2020); and even viral divergences such as SARS-CoV-2 virus (Rambaut et al. 2020; Holmes et al. 2021). The two major releases of IQ-TREE (Nguyen et al. 2015; Minh et al. 2020b) have been cited more than 44,000 times (Google Scholar; 17th March 2026) and downloaded more than 1.3 million times (combined data from the GitHub repository and Bioconda package). IQ-TREE is also an integral component of many open-source biomedical applications such as CIPRES (Miller et al. 2010), GTDB (Parks et al. 2022), QIIME2 (Bolyen et al. 2019), OrthoFinder (Emms and Kelly 2019), Nextstrain (Hadfield et al. 2018), Galaxy (Galaxy Community 2024), PhyloSuite (Zhang et al. 2020; Xiang et al. 2023), TBtools (Chen et al. 2023), and Get_PhyloMarkers (Vinuesa et al. 2018).

Since the release of IQ-TREE version 2 in 2020, we have substantially expanded its capabilities, allowing users to perform data analyses under additional models and scenarios. Here, we introduce IQ-TREE version 3 and highlight its key developments, including mixture models, concordance factors, sequence simulation, protein model estimation, divergence time estimation, and pathogen tree inference.

## Mixture models

Phylogenetic analyses are often conducted with partitioned models, which assign different substitution models to different a priori defined groups of sites to account for heterogeneous evolutionary processes along genomes and/or within genes (e.g. among codon positions). Partitioned models are available in RAxML (Stamatakis 2014), RAxML-NG (Kozlov et al. 2019), and IQ-TREE 2 (Minh et al. 2020b); and tools like PartitionFinder (Lanfear et al. 2017) can merge partitions to reduce over-parameterization.

IQ-TREE 2 implemented mixture models (Le et al. 2008), which assume a user-defined number (*k*) of substitution models (or classes), like partition models. However, unlike partition models, users do not need to specify how to partition the data in mixture models. Rather, model-based probabilities of sites are conditional upon classes, and each site has a certain probability (or weight) of belonging to each class. The site likelihood is calculated as the weighted average of the conditional probabilities, giving the marginal probability of the site pattern. Models of rate heterogeneity among sites based on a Gamma distribution (Yang 1994) or free-rates (Soubrier et al. 2012; Kalyaanamoorthy et al. 2017) are versions of mixture models. Mixture models in IQ-TREE 2 have been used to address many deep phylogenetic questions (e.g. Redmond and McLysaght 2021; Williamson et al. 2025). In particular, such models help to avoid artefacts such as long-branch attraction (Felsenstein 1978; Wang et al. 2018; Leuchtenberger et al. 2020), which can be exacerbated with simpler models.

IQ-TREE 3 substantially improves the IQ-TREE 2 implementation by automatically determining the number of classes (*k*) and the substitution models for each class via the MixtureFinder algorithm (Ren et al. 2025) for DNA data. In this way, the usage of DNA mixture models is now even simpler than partitioned models because no partition file is needed. This also removes user-subjectivity in determining the initial partitioning scheme, although it does come at the cost of additional computational resources: the amount of time and memory required by mixture models increases linearly with *k*. For protein data, in addition to profile mixtures (Wang et al. 2008) and structure-based mixture models (Le et al. 2008) available in version 2, IQ-TREE 3 allows users to estimate amino-acid exchangeability matrices under complex profile mixture models, the so-called GTRpmix model (Baños et al. 2024). Notably, IQ-TREE 3 can also relax the assumption of a single tree common to many phylogenetic analyses by using the Mixtures Across Sites and Trees (MAST) model (Wong et al. 2024), which generalizes the General Heterogeneous evolution On a Single Topology (GHOST) mixture model of branch lengths (Crotty et al. 2020). The MAST model can be useful to examine controversies between competing phylogenetic hypotheses and to pinpoint which sites in an alignment are best explained by which topologies; currently, it requires a user-provided set of input tree topologies.

## Concordance factors

While the MAST model can summarize the contributions of a limited set of alternative tree topologies, IQ-TREE 3 also allows users to summarize discordance on all branches of a reference tree (e.g. an estimate of the species tree). We previously introduced methods to calculate two measures, called the gene concordance factor (gCF) and the site concordance factor (sCF) (Minh et al. 2020a), which quantify the level of concordance between each branch of the reference tree and the genes/loci (for the gCF) and sites (for the sCF). As the original sCF is based on parsimony and can be affected by homoplasy (similarities due to processes other than common ancestry) and taxon sampling, we introduced a likelihood-based version of the sCF (Mo et al. 2023) that partially overcomes these issues. All of these methods and related summary statistics are available in IQ-TREE 3.

## Sequence simulations

Sequence simulations are used in many scenarios, such as evaluating new methods, testing hypotheses with parametric bootstrap (Giacomelli et al. 2025), performing approximate Bayesian computation (Csilléry et al. 2010), and generating training and testing data to help with the development of machine learning applications (Smith and Hahn 2023, but see Trost et al. 2024). However, existing tools like Seq-Gen (Rambaut and Grassly 1997), Dawg (Cartwright 2005), and INDELible (Fletcher and Yang 2009) lack support for mixture and other complex models. IQ-TREE 3 implements a built-in alignment simulator called AliSim (Ly-Trong et al. 2022) to take advantage of all advanced models available in IQ-TREE. To optimize speed, AliSim provides two different algorithms, one to simulate sequences of low divergence and another for high divergence, and can adaptively determine which algorithm to use. AliSim substantially outperforms Seq-Gen, Dawg, and INDELible in terms of both runtimes and memory usage. We also provided a high-performance version, AliSim-HPC (Ly-Trong et al. 2023), which can parallelize the simulations over multicore CPUs and multinode clusters.

## New protein models

Protein data have been used to address many phylogenetic questions, especially at deep time scales. In many cases, analyses rely on empirical protein models like JTT (Jones et al. 1992), WAG (Whelan and Goldman 2001), and LG (Le and Gascuel 2008), which were estimated from large collections of alignments spanning many clades on the tree of life. The scale of modern phylogenomic datasets and powerful computers present an opportunity to estimate models tailored specifically to individual clades. To facilitate this, IQ-TREE 3 provides a new maximum likelihood tool called QMaker (Minh et al. 2021) to estimate a protein substitution matrix for a user-provided set of protein alignments and introduces a general matrix Q.pfam and a series of new clade-specific matrices—Q.bird, Q.insect, Q.mammal, Q.plant, Q.yeast—derived from large empirical datasets (Jarvis et al. 2014; Misof et al. 2014; Ran et al. 2018; Shen et al. 2018; Wu et al. 2018; El-Gebali et al. 2019). These matrices will be useful for phylogenetic analyses of these and related clades. IQ-TREE 3 also provides an extension of QMaker for nonreversible models (Dang et al. 2022) and a series of corresponding nonreversible Q matrices, as well as two new matrices, EAL (Eukaryotes and Archaea) and ELM (Eukaryotes) (Baños et al. 2024), to be used with profile mixture models. The nonreversible matrices allow IQ-TREE 3 to reconstruct rooted trees without the requirement of an outgroup (e.g. Naser-Khdour et al. 2022; Wang and Luo 2025), as well as to infer the confidence of the placement of the root via the rootstrap (Naser-Khdour et al. 2022).

## Pandemic-scale phylogenetics

IQ-TREE 3 integrates CMAPLE (De Maio et al. 2023; Ly-Trong et al. 2024), an ultra-fast method for inferring trees with millions of closely related sequences, such as those produced by global SARS-CoV-2 (the virus that caused the COVID-19 pandemic) sequencing efforts (McBroome et al. 2021). This enables IQ-TREE 3 to perform functions that are useful in tracking large pathogen outbreaks, such as online sample placement. Additionally, IQ-TREE 3 introduces a dedicated “--pathogen” option for dynamically selecting between the CMAPLE and IQ-TREE search algorithms to optimize runtime based on sequence divergence: CMAPLE is selected for highly similar sequences, and IQ-TREE is selected for more distantly related sequences.

Moreover, IQ-TREE 3 and CMAPLE implement subtree pruning and regrafting tree assessment (SPRTA), a fast and appropriate approach for assessing phylogenetic uncertainty in genomic epidemiology (De Maio et al. 2025). SPRTA can be invoked via the “--sprta” option.

## Time tree estimation

IQ-TREE 3 provides two facilities for divergence time estimation. The first is the Least Squares Dating method, LSD2 (To et al. 2016), which is suitable for dating very large phylogenies with tip date information, such as viral phylogenies. The second is the new IQ2MC workflow (Demotte et al. 2025) to facilitate Bayesian divergence time estimation with MCMCTree (dos Reis and Yang 2011). In the IQ2MC workflow, IQ-TREE 3 is used to compute the gradient vector and Hessian matrix of the branch lengths on a fixed tree topology, which are then used for fast approximate Bayesian MCMC sampling with MCMCTree. IQ-TREE dramatically speeds up the precalculation steps needed by MCMCTree, particularly for large phylogenomic datasets, and allows the use of the wide range of IQ-TREE's complex substitution models (such as mixture models), which were previously unavailable for time tree inference, and which are essential for dating ancient phylogenies.

## Other advances

Finally, IQ-TREE 3 also includes a reimplementation of fast distance-based methods such as Neighbor-Joining (Saitou and Nei 1987) via the DecentTree library (Wang et al. 2023). It also includes a Python interface through piqtree (McArthur et al. 2026), an open-source Python package that exposes IQ-TREE's phylogenetic inference engine as a library of Python functions, thus facilitating interactive analyses in environments such as Jupyter notebooks and simplifying the development of custom phylogenetic workflows.

## Conclusions

IQ-TREE version 3 presents a joint collaborative effort among researchers from across the globe, which substantially extends version 2. Particularly, we would like to advocate for the use of mixture models instead of only using partition models, especially as the single-model assumption within a locus is often violated (Ren et al. 2025). In addition, if the single-tree assumption common to concatenation approaches is violated, then users should employ the MAST model and/or interrogate their data by examining concordance factors (Lanfear and Hahn 2024).

We strive to continuously support the user community. Every new feature is accompanied by a tutorial and the manual is frequently updated at https://iqtree.github.io/doc/. User questions, issue reports, and feature requests can be submitted at the GitHub repository.

## Data Availability

There are no data with this paper.
